# Supplementary material for: Retrospective Genotyping of Enteroviruses Using a Diagnostic Nanopore Sequencing Workflow
Source: Pathogens. 2024 May 8;13(5):390. doi: 10.3390/pathogens13050390 (PMC11124337; doi:10.3390/pathogens13050390)
Supplement: Supplementary file 1 [file pathogens-13-00390-s001.zip › pathogens-2951102-supplementary.pdf]

Supplementary Material

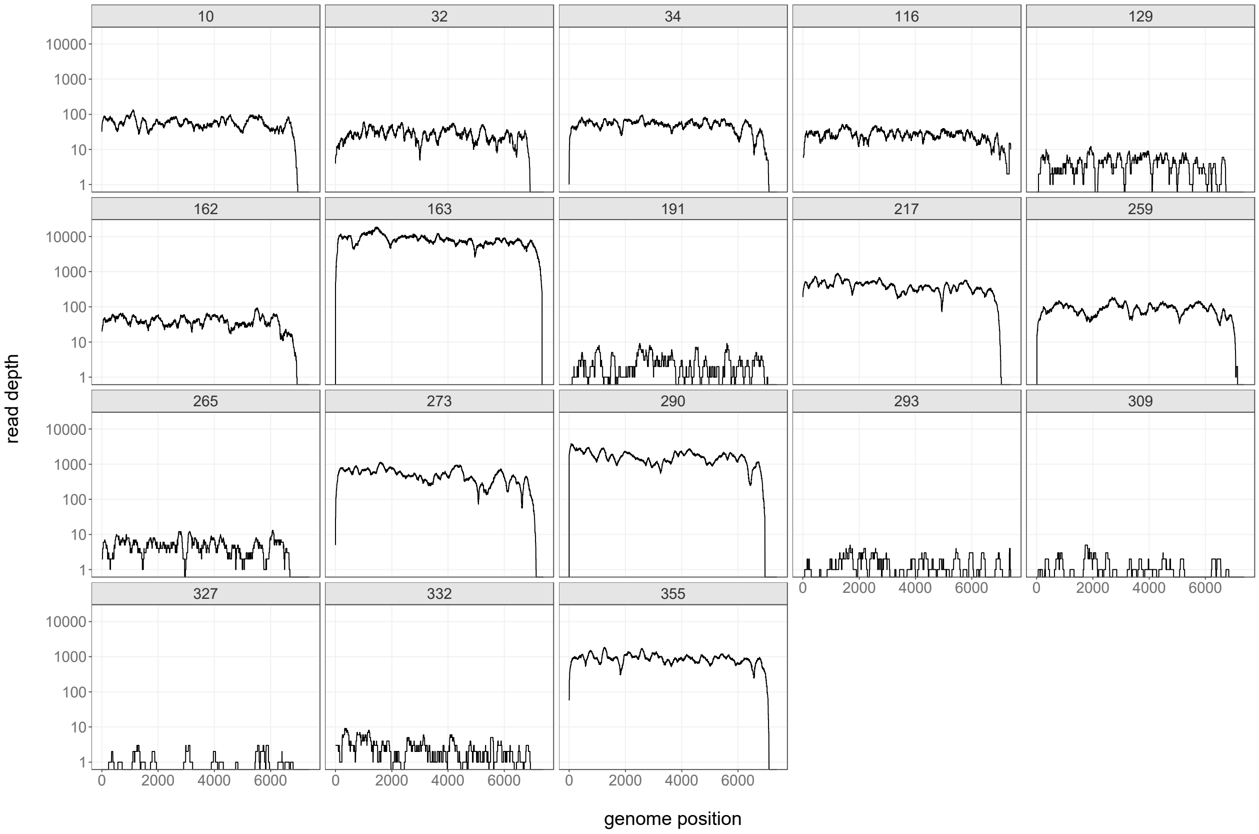

Supplementary Figure S1. Coverage plots of 18 samples sequenced using an untargeted whole genome approach.

Supplementary Table S1. Patient and sample characteristics, sequencing and qPCR results of all analyzed samples. N/A in the column Species refers to unsuccessful genotyping. N/A in the qPCR columns indicates undetectable viral loads.

#	Study number	Host age	Sampling Date YYYY/MM	material_class	Season respiratory	Species	Genotype	Double infections	qPCR ct_entero	qPCR ct_rhino
**1**	1	67	2019/09	Respiratory specimen	2019/2020	Rhinovirus B	RV-B14		NA	28.24
**2**	3	26	2019/09	Respiratory specimen	2019/2020	Rhinovirus A	RV-A67		NA	21.59
**3**	4	67	2019/09	Respiratory specimen	2019/2020	Rhinovirus A	RV-A21		NA	28.32
**4**	5	62	2019/09	Respiratory specimen	2019/2020	Rhinovirus A	RV-A33		NA	27.5
**5**	6	47	2019/09	Respiratory specimen	2019/2020	Rhinovirus A	RV-A71		NA	23.68
**6**	7	23	2019/09	Respiratory specimen	2019/2020	Rhinovirus B	RV-B86		NA	28
**7**	8	46	2019/09	Respiratory specimen	2019/2020	Rhinovirus A	RV-A34		NA	31.25
**8**	9	18	2019/09	Respiratory specimen	2019/2020	Rhinovirus B	RV-B6		NA	29.4
**9**	10	59	2019/09	Respiratory specimen	2019/2020	Rhinovirus C	RV-C45		NA	28.07
**10**	11	50	2019/10	Respiratory specimen	2019/2020	Rhinovirus B	RV-B3		NA	29.38
**11**	12	62	2019/10	Respiratory specimen	2019/2020	Rhinovirus B	RV-B70		NA	23.43
**12**	13	45	2019/10	Respiratory specimen	2019/2020	Rhinovirus B	RV-B6		NA	29.38
**13**	15	61	2019/10	Respiratory specimen	2019/2020	Rhinovirus B	RV-B35		33.55	26.8

**14**	16	27	2019/10	Respiratory specimen	2019/2020	Rhinovirus A	RV-A2	NA	31.06
**14**	17	52	2019/10	Respiratory specimen	2019/2020	Rhinovirus A	RV-A1	NA	27.21
**16**	20	58	2019/10	Respiratory specimen	2019/2020	Rhinovirus A	RV-A49	NA	31.02
**17**	22	59	2019/11	Respiratory specimen	2019/2020	Rhinovirus A	RV-A34	NA	27.37
**18**	23	42	2019/11	Respiratory specimen	2019/2020	Rhinovirus A	RV-A1	NA	28.67
**19**	25	57	2019/11	Respiratory specimen	2019/2020	Rhinovirus B	RV-B14	NA	20.85
**20**	26	51	2019/11	Respiratory specimen	2019/2020	Rhinovirus A	RV-A80	NA	24.66
**21**	27	16	2019/11	Respiratory specimen	2019/2020	Rhinovirus B	RV-B3	NA	29.87
**22**	29	31	2019/11	Respiratory specimen	2019/2020	Rhinovirus B	RV-B3	NA	32.35
**23**	32	46	2019/12	Respiratory specimen	2019/2020	Rhinovirus B	unclassified	NA	27.73
**24**	34	60	2019/12	Respiratory specimen	2019/2020	Rhinovirus C	RV-C42	32.96	27.74
**25**	36	46	2019/12	Respiratory specimen	2019/2020	Rhinovirus A	RV-A1	NA	27.81
**26**	38	55	2019/12	Respiratory specimen	2019/2020	Rhinovirus A	RV-A32	36.08	24.26
**27**	39	52	2019/12	Respiratory specimen	2019/2020	Rhinovirus A	RV-A15	NA	25.1
**28**	40	39	2019/12	Respiratory specimen	2019/2020	Rhinovirus B	RV-B14	NA	27.39
**29**	41	89	2020/01	Respiratory specimen	2019/2020	Rhinovirus A	RV-A11	NA	34.09
**30**	43	69	2020/01	Respiratory specimen	2019/2020	Rhinovirus A	RV-A11	NA	25.66
**31**	51	61	2020/01	Respiratory specimen	2019/2020	Rhinovirus A	RV-A47	NA	23.29
**32**	53	67	2020/02	Other	NA	Rhinovirus A	RV-A20	NA	32.18
**33**	55	35	2020/02	Respiratory specimen	2019/2020	Rhinovirus A	RV-A53	NA	29.05
**34**	56	63	2020/02	Respiratory specimen	2019/2020	Rhinovirus A	RV-A34	NA	27.53
**35**	60	76	2020/02	Respiratory specimen	2019/2020	Rhinovirus A	RV-A24	NA	25.29
**36**	64	17	2020/03	Respiratory specimen	2019/2020	Rhinovirus B	RV-B27	NA	32.22
**37**	65	27	2020/03	Respiratory specimen	2019/2020	Rhinovirus A	RV-A60	NA	19.63
**38**	69	39	2020/03	Respiratory specimen	2019/2020	Rhinovirus A	RV-A46	NA	17.96
**39**	70	35	2020/03	Respiratory specimen	2019/2020	Rhinovirus A	RV-A31	NA	23.58
**40**	71	76	2020/03	Respiratory specimen	2019/2020	Rhinovirus C	RV-C24	28.96	23.33
**41**	73	0	2019/07	Stool	NA	Enterovirus A	EV-A71	29.01	NA
**42**	74	1	2019/07	Stool	NA	Enterovirus B	CVB5	24.68	NA
**43**	75	1	2019/07	Stool	NA	Enterovirus A	CVA10	22.68	40.72
**44**	76	0	2019/07	Stool	NA	Enterovirus A	EV-A71	33.04	NA
**45**	77	37	2019/07	CSF	NA	Enterovirus B	E25	34.31	NA
**46**	78	0	2019/07	Stool	NA	Enterovirus B	CVB5	30.73	NA
**47**	79	37	2019/07	CSF	NA	Enterovirus B	CVB5	31.22	NA
**48**	80	0	2019/07	Stool	NA	Enterovirus B	CVB5	32.54	NA
**49**	81	0	2019/09	CSF	NA	Enterovirus B	CVB5	33.67	NA
**50**	82	1	2019/09	Stool	NA	Enterovirus B	E6	NA	NA
**51**	84	1	2019/09	Stool	NA	Enterovirus A	CVA6	22.49	38.1
**52**	86	30	2019/12	Stool	NA	Enterovirus B	E20	34.75	NA
**53**	87	0	2019/12	Stool	NA	Enterovirus B	E7	35.32	27.54
**54**	88	0	2019/12	Stool	NA	Enterovirus B	CVB4	NA	NA
**55**	90	0	2019/12	CSF	NA	Enterovirus B	E7	NA	NA
**56**	91	2	2019/12	Stool	NA	Enterovirus B	E20	28.8	NA
**57**	92	0	2019/12	Stool	NA	Enterovirus A	CVA6	27.1	NA
**58**	93	0	2019/12	Stool	NA	Enterovirus A	CVA6	28.59	NA
**59**	94	36	2020/01	Stool	NA	Enterovirus B	E7	36.97	NA
**60**	95	0	2020/05	Stool	NA	Enterovirus B	E21	22.48	33.12
**61**	96	52	2020/11	CSF	NA	Enterovirus B	E30	NA	NA
**62**	98	0	2020/04	Stool	NA	Enterovirus B	B27	NA	31.31

**63**	100	0	2022/10	Stool	NA	Enterovirus B	CVA9		24.79	NA
**64**	101	0	2022/10	Stool	NA	Enterovirus B	E18		32.53	NA
**65**	102	0	2022/10	CSF	NA	Enterovirus B	E18		38.32	NA
**66**	104	0	2022/12	Stool	NA	Enterovirus B	CVA9		31.71	39.54
**67**	105	4	2022/12	Stool	NA	Enterovirus B	CVB3		NA	NA
**68**	106	1	2021/08	Stool	NA	Enterovirus A	CVA10		22.85	NA
**69**	107	0	2021/09	Stool	NA	Enterovirus B	CVB4		34.82	NA
**70**	108	0	2021/09	CSF	NA	Enterovirus B	CVB4		24.41	NA
**71**	109	0	2021/09	Stool	NA	Enterovirus B	CVB3		26.68	NA
**72**	110	5	2021/09	Stool	NA	Rhinovirus A	RV-A53		NA	29.15
**73**	111	1	2021/11	Stool	NA	Enterovirus B	E6		33.92	NA
**74**	112	0	2021/12	Stool	NA	Enterovirus B	CVB3		29.95	NA
**75**	113	73	2021/12	CSF	NA	Enterovirus A	CVA6		28.69	NA
**76**	116	0	2022/05	CSF	NA	Enterovirus D	EV-D68	Norovirus GII	31.3	NA
**77**	117	0	2022/06	CSF	NA	Enterovirus B	E11		40.93	NA
**78**	118	6	2021/07	CSF	NA	Enterovirus B	E6		35.11	NA
**79**	119	0	2021/07	Stool	NA	Enterovirus A	CVA4		NA	NA
**80**	120	0	2021/08	Stool	NA	Enterovirus B	CVB3		27.6	35.07
**81**	121	0	2021/08	Stool	NA	Enterovirus A	CVA6		26.58	NA
**82**	123	49	2021/08	CSF	NA	Enterovirus B	CVB3		NA	NA
**83**	124	0	2022/07	Stool	NA	Enterovirus B	CVB5		24.87	NA
**84**	125	0	2022/07	CSF	NA	Enterovirus B	CVB5		33.25	NA
**85**	126	0	2022/07	Stool	NA	Enterovirus B	E25		30.26	NA
**86**	127	0	2022/07	CSF	NA	Enterovirus B	CVB5		35.05	NA
**87**	128	0	2022/07	Stool	NA	Enterovirus B	CVB2		23.45	NA
**88**	129	31	2022/08	CSF	NA	Enterovirus B	CVB4		35.64	NA
**89**	130	0	2022/08	Stool	NA	Enterovirus B	CVB1		29.24	NA
**90**	131	1	2022/08	Stool	NA	Enterovirus A	CVA10		21.99	NA
**91**	132	71	2019/09	Respiratory specimen	2019/2020	Rhinovirus A	RV-A25		NA	35.02
**92**	134	56	2019/09	Respiratory specimen	2019/2020	Rhinovirus A	RV-A1		37.63	20.87
**93**	135	73	2019/09	Respiratory specimen	2019/2020	Rhinovirus A	RV-A82		NA	22.77
**94**	137	15	2019/09	Respiratory specimen	2019/2020	Enterovirus B	CVB3		NA	NA
**95**	139	71	2019/09	Respiratory specimen	2019/2020	Rhinovirus C	RV-C41		NA	33.4
**96**	140	25	2019/09	Respiratory specimen	2019/2020	Rhinovirus B	RV-B14		NA	28.77
**97**	141	47	2019/09	Respiratory specimen	2019/2020	Rhinovirus A	RV-A22		NA	25.87
**98**	142	41	2019/09	Respiratory specimen	2019/2020	Rhinovirus B	RV-B3		NA	28.38
**99**	143	2	2019/09	Respiratory specimen	2019/2020	Rhinovirus C	RV-C43		26.5	19.11
**100**	144	69	2019/09	Respiratory specimen	2019/2020	Rhinovirus A	RV-A47		NA	30.71
**101**	145	53	2019/09	Respiratory specimen	2019/2020	Rhinovirus B	RV-B70		NA	22.66
**102**	146	58	2019/09	Respiratory specimen	2019/2020	Rhinovirus A	RV-A1		33.04	15.89
**103**	151	16	2019/10	Respiratory specimen	2019/2020	Rhinovirus B	RV-B27		NA	29.71
**104**	152	27	2019/10	Respiratory specimen	2019/2020	Rhinovirus B	RV-B70		NA	29.87
**105**	153	33	2019/10	Respiratory specimen	2019/2020	Rhinovirus B	RV-B14		NA	32.94
**106**	154	83	2019/10	Respiratory specimen	2019/2020	Rhinovirus A	RV-A1		NA	22.1
**107**	155	28	2019/10	Respiratory specimen	2019/2020	Rhinovirus B	RV-B3		NA	28.67
**108**	156	21	2019/10	Respiratory specimen	2019/2020	Rhinovirus A	RV-A55		NA	18.96
**109**	157	55	2019/10	Respiratory specimen	2019/2020	Rhinovirus A	RV-A57		NA	24.81
**110**	158	60	2019/10	Respiratory specimen	2019/2020	Rhinovirus B	RV-B69		NA	32.67
**111**	160	3	2019/10	Respiratory specimen	2019/2020	Rhinovirus B	RV-B48		NA	25.01
**112**	162	43	2019/10	Respiratory specimen	2019/2020	Rhinovirus C	RV-C1		NA	26.36
**113**	163	78	2019/10	Respiratory specimen	2019/2020	Enterovirus D	EV-D68		24.56	31.13

**114**	166	2	2019/11	Respiratory specimen	2019/2020	Rhinovirus B	RV-B91		NA	27.42
**115**	168	36	2019/11	Respiratory specimen	2019/2020	Rhinovirus A	RV-A9		NA	21.6
**116**	172	55	2019/11	Respiratory specimen	2019/2020	Rhinovirus B	RV-B26		NA	25.88
**117**	175	55	2019/11	Respiratory specimen	2019/2020	Rhinovirus A	RV-A100		NA	27.94
**118**	177	58	2019/11	Respiratory specimen	2019/2020	Rhinovirus A	RV-A66		NA	35.12
**119**	179	27	2019/11	Respiratory specimen	2019/2020	Rhinovirus C	RV-C41		NA	23.09
**120**	183	72	2019/12	Respiratory specimen	2019/2020	Rhinovirus B	RV-B91		NA	27.08
**121**	185	0	2019/12	Respiratory specimen	2019/2020	Rhinovirus A	RV-A8		NA	28.56
**122**	186	0	2019/12	Respiratory specimen	2019/2020	Enterovirus B	CVB2		29.22	19.65
**123**	187	71	2019/12	Respiratory specimen	2019/2020	Rhinovirus A	RV-A56		NA	31.21
**124**	188	6	2019/12	Respiratory specimen	2019/2020	Rhinovirus B	RV-B3	RV-B86	NA	23.6
**125**	189	54	2019/12	Respiratory specimen	2019/2020	Rhinovirus B	RV-B4		NA	26.4
**126**	190	61	2019/12	Respiratory specimen	2019/2020	Rhinovirus C	RV-C11		39.17	20.6
**127**	191	60	2020/01	Respiratory specimen	2019/2020	Rhinovirus C	RV-C15		NA	32.04
**128**	193	77	2020/01	Respiratory specimen	2019/2020	Rhinovirus C	RV-C15		25.8	17.23
**129**	195	29	2020/01	Respiratory specimen	2019/2020	Rhinovirus B	RV-B3		NA	26.05
**130**	196	70	2020/01	Respiratory specimen	2019/2020	Rhinovirus C	RV-C53		NA	NA
**131**	198	51	2020/01	Respiratory specimen	2019/2020	Rhinovirus A	RV-A11		NA	19.76
**132**	200	86	2020/01	Respiratory specimen	2019/2020	Rhinovirus A	RV-A34		NA	22.66
**133**	201	64	2020/02	Respiratory specimen	2019/2020	Rhinovirus A	RV-A39		NA	26.39
**134**	202	64	2020/02	Respiratory specimen	2019/2020	Rhinovirus C	RV-C33		37.87	37.9
**135**	206	58	2020/02	Respiratory specimen	2019/2020	Rhinovirus B	RV-B84		NA	18.87
**136**	207	67	2020/02	Respiratory specimen	2019/2020	Rhinovirus A	RV-A20		NA	23.72
**137**	211	73	2020/02	Respiratory specimen	2019/2020	Rhinovirus A	RV-A12		NA	24.44
**138**	216	3	2020/03	Respiratory specimen	2019/2020	Rhinovirus A	RV-A28		NA	27.84
**139**	217	44	2020/03	Respiratory specimen	2019/2020	Rhinovirus C	RV-C17		35.84	25.02
**140**	219	34	2021/07	Respiratory specimen	2021/2022	Rhinovirus A	RV-A15		NA	17.66
**141**	223	33	2021/07	Respiratory specimen	2021/2022	Rhinovirus A	RV-A24		NA	24.63
**142**	224	63	2021/07	Respiratory specimen	2021/2022	Rhinovirus A	RV-A61		NA	14.99
**143**	225	27	2021/07	Respiratory specimen	2021/2022	Rhinovirus A	RV-A36		29.4	27.66
**144**	226	18	2021/07	Respiratory specimen	2021/2022	Rhinovirus A	RV-A31		NA	28.84
**145**	227	72	2021/07	Respiratory specimen	2021/2022	Rhinovirus B	RV-B69		36.74	19.42
**146**	228	61	2021/07	Respiratory specimen	2021/2022	Rhinovirus A	RV-A24		NA	27.81
**147**	229	1	2021/07	Respiratory specimen	2021/2022	Enterovirus B	CVB3		NA	34.94
**148**	230	49	2021/07	Respiratory specimen	2021/2022	Rhinovirus A	RV-A24		NA	24.86
**149**	231	65	2021/07	Respiratory specimen	2021/2022	Rhinovirus A	RV-A34		NA	35.19
**150**	233	54	2021/08	Respiratory specimen	2021/2022	Rhinovirus A	RV-A25		NA	21

**151**	234	61	2021/08	Respiratory specimen	2021/2022	Rhinovirus A	RV-A31		NA	31.44
**152**	235	23	2021/08	Respiratory specimen	2021/2022	Rhinovirus A	RV-A61		NA	28.99
**153**	237	66	2021/08	Respiratory specimen	2021/2022	Rhinovirus A	RV-A65		NA	25.87
**154**	239	19	2021/08	Respiratory specimen	2021/2022	Rhinovirus B	RV-B27		NA	29.11
**155**	241	1	2021/08	Other	NA	Enterovirus A	CVA6		20.4	41.01
**156**	242	31	2021/08	Respiratory specimen	2021/2022	Rhinovirus A	RV-A47		NA	27.03
**157**	243	29	2021/08	Respiratory specimen	2021/2022	Rhinovirus A	RV-A47		NA	20.06
**158**	244	64	2021/08	Respiratory specimen	2021/2022	Rhinovirus A	RV-A46		NA	16.52
**159**	247	56	2021/08	Respiratory specimen	2021/2022	Rhinovirus A	RV-A24		NA	26.47
**160**	249	50	2021/09	Respiratory specimen	2021/2022	Rhinovirus A	RV-A20		NA	31.72
**161**	250	41	2021/09	Respiratory specimen	2021/2022	Rhinovirus A	RV-A58		NA	24.84
**162**	251	65	2021/09	Respiratory specimen	2021/2022	Enterovirus A	CVA6		34.54	NA
**163**	253	50	2021/09	Respiratory specimen	2021/2022	Rhinovirus A	RV-A24		NA	24.67
**164**	255	54	2021/09	Respiratory specimen	2021/2022	Rhinovirus A	RV-A47		NA	25.04
**165**	256	0	2021/09	Respiratory specimen	2021/2022	Rhinovirus A	RV-A53		NA	22.85
**166**	258	51	2021/09	Respiratory specimen	2021/2022	Rhinovirus B	RV-B42		NA	20.46
**167**	259	4	2021/09	Respiratory specimen	2021/2022	Rhinovirus C	RV-C25		27.86	27.79
**168**	261	0	2021/09	Other	NA	Enterovirus A	CVA6		21.59	42.79
**169**	263	43	2021/09	Respiratory specimen	2021/2022	Rhinovirus A	RV-A80		NA	20.37
**170**	265	7	2021/10	Respiratory specimen	2021/2022	Rhinovirus C	RV-C1		NA	25.35
**171**	266	1	2021/10	Respiratory specimen	2021/2022	Enterovirus B	CVB2	RV-A53	NA	24.2
**172**	267	76	2021/10	Respiratory specimen	2021/2022	Rhinovirus A	RV-A25		NA	23.09
**173**	268	70	2021/10	Respiratory specimen	2021/2022	Rhinovirus A	RV-A24		NA	23.49
**174**	271	42	2021/10	Respiratory specimen	2021/2022	Rhinovirus A	RV-A85		NA	33.2
**175**	272	19	2021/10	Respiratory specimen	2021/2022	Rhinovirus A	RV-A2		NA	30.16
**176**	273	61	2021/10	Respiratory specimen	2021/2022	Rhinovirus A	RV-A78	Bocavirus	32.76	22.52
**177**	274	46	2021/10	Respiratory specimen	2021/2022	Rhinovirus A	RV-A31		NA	24.4
**178**	275	54	2021/10	Respiratory specimen	2021/2022	Rhinovirus A	RV-A29		NA	24.58
**179**	276	36	2021/10	Respiratory specimen	2021/2022	Rhinovirus A	RV-A47		NA	27.88
**180**	277	1	2021/11	Respiratory specimen	2021/2022	Enterovirus A	CVA6		20.31	39.05
**181**	278	32	2021/11	Respiratory specimen	2021/2022	Rhinovirus B	RV-B83		NA	31.69
**182**	281	36	2021/11	Respiratory specimen	2021/2022	Rhinovirus B	RV-B3		NA	31.27
**183**	283	70	2021/11	Respiratory specimen	2021/2022	Rhinovirus C	RV-C11		NA	25.51
**184**	285	64	2021/11	Respiratory specimen	2021/2022	Rhinovirus B	RV-B83		NA	25.57
**185**	286	58	2021/11	Respiratory specimen	2021/2022	Rhinovirus A	RV-A46		31	27.9
**186**	288	53	2021/11	Respiratory specimen	2021/2022	Rhinovirus A	RV-A47		NA	23.81
**187**	289	0	2021/11	Respiratory specimen	2021/2022	Rhinovirus A	RV-A78		43.22	19.44
**188**	290	66	2021/11	Respiratory specimen	2021/2022	Rhinovirus C	RV-C1		NA	19.79

**189**	293	2	2021/12	Respiratory specimen	2021/2022	Enterovirus D	EV-D68	40.55	NA
**190**	294	51	2021/12	Respiratory specimen	2021/2022	Rhinovirus A	RV-A47	NA	27.45
**191**	302	43	2021/12	Respiratory specimen	2021/2022	Rhinovirus C	RV-C51	NA	39.36
**192**	303	41	2021/12	Respiratory specimen	2021/2022	Rhinovirus A	RV-A47	NA	25.32
**193**	304	0	2021/12	Respiratory specimen	2021/2022	Rhinovirus A	RV-A57	NA	28.31
**194**	305	1	2021/12	Respiratory specimen	2021/2022	Enterovirus A	CVA6	23.21	43.03
**195**	309	64	2022/01	Respiratory specimen	2021/2022	Rhinovirus B	RV-B69	NA	29.11
**196**	310	49	2022/01	Respiratory specimen	2021/2022	Rhinovirus C	RV-C56	NA	27.7
**197**	313	52	2022/01	Respiratory specimen	2021/2022	Rhinovirus A	RV-A15	NA	25.1
**198**	321	68	2022/02	Respiratory specimen	2021/2022	Rhinovirus A	RV-A59	NA	NA
**199**	324	70	2022/02	Respiratory specimen	2021/2022	Rhinovirus A	RV-A46	NA	29.31
**200**	325	26	2022/02	Respiratory specimen	2021/2022	Rhinovirus A	RV-A59	NA	25.29
**201**	327	65	2022/02	Respiratory specimen	2021/2022	Rhinovirus C	RV-C1	NA	32.74
**202**	331	55	2022/03	Respiratory specimen	2021/2022	Rhinovirus A	RV-A31	NA	35.98
**203**	332	59	2022/03	Respiratory specimen	2021/2022	Rhinovirus C	RV-C1	NA	24.61
**204**	333	1	2022/03	Other	NA	Enterovirus A	CVA6	18.93	39.38
**205**	334	34	2022/03	Respiratory specimen	2021/2022	Rhinovirus C	RV-C5	NA	27.49
**206**	337	61	2022/03	Respiratory specimen	2021/2022	Rhinovirus A	RV-A85	NA	26.29
**207**	338	68	2022/03	Respiratory specimen	2021/2022	Rhinovirus A	RV-A11	NA	25.29
**208**	340	70	2022/03	Respiratory specimen	2021/2022	Rhinovirus A	RV-A24	NA	33.92
**209**	341	1	2022/03	Respiratory specimen	2021/2022	Enterovirus A	CVA6	21.62	39.45
**210**	343	42	2022/04	Respiratory specimen	2021/2022	Rhinovirus A	RV-A58	NA	28.52
**211**	345	31	2022/04	Respiratory specimen	2021/2022	Enterovirus A	CVA10	33.63	NA
**212**	346	55	2022/04	Respiratory specimen	2021/2022	Enterovirus A	CVA10	NA	NA
**213**	347	66	2022/04	Respiratory specimen	2021/2022	Enterovirus A	CVA10	NA	36.18
**214**	348	69	2022/04	Respiratory specimen	2021/2022	Enterovirus A	CVA6	NA	29.91
**215**	349	36	2022/04	Respiratory specimen	2021/2022	Rhinovirus C	C45	NA	NA
**216**	351	70	2022/04	Respiratory specimen	2021/2022	Enterovirus B	CVB3	NA	21.8
**217**	355	0	2022/04	Respiratory specimen	2021/2022	Rhinovirus C	C42	28.4	22.66
**218**	358	1	2022/07	Other	NA	NA	NA	18.9	41.53
**219**	361	56	2019/12	Respiratory specimen	2019/2020	Rhinovirus C	RV-C26	NA	27.38
**220**	362	0	2020/02	Respiratory specimen	2019/2020	Rhinovirus B	RV-B72	NA	36.69
**221**	363	73	2022/03	CSF	NA	Rhinovirus C	RV-C11	NA	NA
**222**	364	53	2020/02	Respiratory specimen	2019/2020	Rhinovirus A	RV-A12	NA	30.02
**223**	365	36	2021/09	Respiratory specimen	2021/2022	Rhinovirus A	RV-A36	NA	NA
**224**	367	0	2021/11	Respiratory specimen	2021/2022	NA	NA	NA	22.86
**225**	369	78	2019/10	Respiratory specimen	2019/2020	NA	NA	NA	19.21
**226**	370	15	2019/12	Respiratory specimen	2019/2020	NA	NA	NA	26.59
**227**	371	76	2019/12	Respiratory specimen	2019/2020	Rhinovirus A	RV-A36	NA	30.95

**228**	373	63	2020/02	Respiratory specimen	2019/2020	NA	NA		36.57	27.24
**229**	374	58	2020/03	Respiratory specimen	2019/2020	NA	NA		NA	27.87
**230**	375	0	2022/05	Stool	NA	NA	NA		27.76	NA
**231**	376	9	2019/09	Respiratory specimen	2019/2020	NA	NA		NA	26.66
**232**	380	63	2019/10	Respiratory specimen	2019/2020	NA	NA		NA	29.88
**233**	382	69	2019/11	Respiratory specimen	2019/2020	Rhinovirus C	RV-C12		30	19.66
**234**	383	75	2019/11	Respiratory specimen	2019/2020	NA	NA		NA	28.59
**235**	384	68	2019/11	Respiratory specimen	2019/2020	NA	NA		NA	22.67
**236**	386	68	2019/12	Respiratory specimen	2019/2020	Rhinovirus C	RV-C43		33.53	26.87
**237**	387	57	2019/12	Respiratory specimen	2019/2020	NA	NA		NA	26.43
**238**	388	6	2020/01	Respiratory specimen	2019/2020	NA	NA		37.19	25.02
**239**	389	5	2020/02	Respiratory specimen	2019/2020	Rhinovirus A	RV-A78		NA	22.61
**240**	390	0	2020/02	Respiratory specimen	2019/2020	Rhinovirus C	RV-C7		35.1	26.72
**241**	391	0	2020/02	Respiratory specimen	2019/2020	NA	NA		NA	26.93
**242**	392	3	2020/03	Respiratory specimen	2019/2020	NA	NA		NA	29
**243**	393	50	2021/07	Respiratory specimen	2021/2022	Rhinovirus A	RV-A24		NA	24.39
**244**	398	20	2021/12	Respiratory specimen	2021/2022	NA	NA		NA	28.15
**245**	399	79	2021/12	Respiratory specimen	2021/2022	Rhinovirus A	RV-A20		NA	21.75
**246**	401	52	2022/02	Respiratory specimen	2021/2022	Rhinovirus A	RV-A25		NA	27.79
**247**	402	69	2022/02	Respiratory specimen	2021/2022	Rhinovirus A	RV-A15		NA	27.59
**248**	403	24	2022/02	Respiratory specimen	2021/2022	NA	NA		NA	29.69
**249**	405	42	2022/03	Respiratory specimen	2021/2022	Rhinovirus A	RV-A30		NA	32.19
**250**	406	28	2022/04	Respiratory specimen	2021/2022	Rhinovirus A	RV-A58	RV-A59	NA	29.68
**251**	407	58	2022/04	Respiratory specimen	2021/2022	Rhinovirus B	RV-B72		NA	26.68
**252**	408	50	2022/04	Respiratory specimen	2021/2022	Rhinovirus A	RV-A12		NA	25.62
**253**	409	0	2022/04	Respiratory specimen	2021/2022	Rhinovirus A	RV-A36		NA	27.81
**254**	411	52	2022/04	Respiratory specimen	2021/2022	NA	NA		NA	29.32
**255**	412	37	2022/06	Other	NA	Enterovirus A	CVA10		30.38	NA
